# Digital Pathology Allows for Global Second Opinions for Urologic Malignancies

**DOI:** 10.1007/s11934-025-01255-7

**Published:** 2025-02-08

**Authors:** Daniel J. Shepherd, Jennifer B. Gordetsky

**Affiliations:** 1 Department of Pathology, Microbiology, and Immunology, Vanderbilt University Medical Center, 445 Great Circle Road, Nashville, TN 37228 USA; 2 Department of Urology, Vanderbilt University Medical Center, Nashville, TN USA

**Keywords:** Telepathology, Digital pathology, Second opinion, Developing countries

## Abstract

**Purpose of Review:**

Digital pathology, the use of digital images for histopathologic diagnosis, is transforming the practice of pathology. This review discusses the ability of digital pathology to assist with second opinions for challenging cases in genitourinary pathology worldwide.

**Recent Findings:**

While traditional pathology is limited by physical hardware such as microscopes and glass slides, digital pathology creates opportunities for the rapid sharing of diagnostic materials with colleagues and experts worldwide. This technology can greatly facilitate sharing challenging cases from low-resource areas where pathology services or subspecialty expertise are not available.

**Summary:**

As the incidence of kidney, prostate, and testicular cancer continues to increase in both high-income and developing countries, digital pathology may be the solution for expert opinions in diagnosing urologic disease worldwide.

## Introduction

Digital pathology, the use of digital images for histopathologic diagnosis, is transforming the practice of pathology. While traditional pathology is limited by physical hardware such as microscopes and glass slides, digital pathology creates opportunities for the rapid sharing of diagnostic materials with colleagues and experts worldwide. Importantly, it can greatly facilitate sharing challenging cases from low-resource areas where there may be no pathologists available or a pathologist with subspecialty expertise is lacking. This practice of second opinion consultation via telepathology therefore has the potential to improve patient care on a global scale, especially in areas challenged by limited resources.

The age-standardized incidence of kidney, prostate, and testicular cancer continues to increase in both high-income and developing countries, while new bladder cancer diagnoses increased 1.5-fold from 1990 to 2013 [1, 2]. Prostate cancer now outpaces lung cancer as the most common cancer diagnosis in men across 112 countries [2]. New classifications, changes in grading, and detailed reporting requirements continue to create challenges in the diagnosis and reporting of genitourinary malignancies. In addition, some genitourinary malignancies such as testicular germ cell tumors are infrequent and therefore rarely encountered outside of the academic setting. These tumors can cause diagnostic difficulty for the generalist practicing pathologist and providing a correct subclassification is critical for the appropriate clinical management [3]. In addition, histologic mimickers of cancer and small cancer foci, especially in bladder and prostate pathology, can create false positive and missed cancer diagnoses. Having a mechanism for reliable access to subspecialty expertise or having access to technology such as artificial intelligence (AI) that can assist in diagnostic dilemmas could be useful in many hospital settings both in the United States (US) and abroad.

### Current Practice

The standard of care in most pathology settings in the US continues to be traditional histopathology. In this method, resected tissue is submitted for gross examination and processing. Tissue is typically fixed in 10% buffered formalin and then processed through a series of alcohols. Once the tissue is processed, it is then embedded in paraffin and cut into 3 to 5 micron-thick slices, which are placed on a glass slide. The tissue on the slide then undergoes de-paraffinization and staining with hematoxylin and eosin (H&E). Most diagnoses rely on interpretation of the findings seen on the H&E-stained glass slide under the microscope. Ancillary studies, such as immunohistochemistry (IHC), special stains, and molecular diagnostics may also be performed on the tissue remaining in the paraffin block. These generate additional glass slides to be reviewed microscopically in the context of the H&E findings.

Similar to subspecialist referral in clinical fields, subspecialty pathologist consultation may be requested by practicing general or community pathologists on challenging cases. After recognizing the need for expert subspecialist consultation, a pathologist will initiate the consultation by mailing the glass slides, printouts of relevant clinical history and radiology studies, and a representative tissue block or unstained slides for further workup to the consultant. Once received, these materials are accessioned by the consultant’s pathology department and delivered to the consultant for formal interpretation in their laboratory information system (LIS). Once an interpretation has been rendered, the original glass slides are returned to the referring institution. As different hospitals utilize different electronic medical record systems, results often must be faxed back to the sending institution to be uploaded into their own system. This process relies on manual labor and requires handling at multiple locations during shipping. Common issues encountered include shipping delays, damage to tissue blocks/glass slides, and loss of potentially irreplaceable materials. Paraffin blocks in particular can be compromised by extreme heat during shipping.

### Second Opinions in GU Pathology

In urologic oncology, critical management decisions can hinge on subtle histopathologic findings, such as muscularis propria invasion in bladder cancer and accurate interpretation of Gleason scores in prostate cancer. The importance of accurate histopathologic diagnosis underscores the potential value of case review by subspecialist GU pathologists. Indeed, numerous studies have documented the potential for significant discordance between original diagnosis and the diagnoses made after subspecialist GU pathologist review. For example, Lee and colleagues found that TURBT diagnoses and staging by subspecialist GU pathologists were significantly discordant with the original interpretation in 26–33% of cases. It was estimated that management would have been impacted in 28% of these cases based on pathology review [4]. Other studies found similar discrepancy rates in TURBT and prostatic urethral biopsy specimens, particularly regarding muscularis propria invasion, stage, and the recognition of clinically important histologic variants of urothelial carcinoma [5, 6]. Larger resection specimens seem to be less prone to clinically significant differences in interpretation [6]. For prostate biopsies, Nguyen et al.. identified a 44% rate of disagreement regarding Gleason score, which would have changed management in a total of 10% of the study patients at the time of publication [7]. Siedow and colleagues found that nearly 20% of their patients were assigned a different NCCN risk group after secondary pathology review of prostate biopsies [8]. In orchiectomy specimens for testicular cancer, Sharma et al.. found clinically significant discrepancies in pathologic findings in about 7% of cases, most commonly a change in lymphovascular invasion status for non-seminomatous germ cell tumors with the potential to impact therapy [9]. An important caveat is that some of these discordances may be explained by interobserver variability inherent in the interpretation of biopsy specimens. However, pathology review at large tertiary care centers can help mitigate this variability through sharing cases with additional subspecialty-trained colleagues to reach consensus, which commonly occurs.

### Digital Pathology for Histopathologic Diagnosis

Digital pathology can be broadly defined as the generation of digitized computer images from conventional glass microscope slides, followed by all downstream steps involved in storing, reviewing, and analyzing those images for diagnosis, research, and education [10]. “Telepathology” refers to the sharing of these digital images across physical space for viewing on a screen [11]. Telepathology encompasses several methods, each with their own set of practical considerations (Table 1). In static telepathology, digital images are captured from regions of interest on a slide and shared remotely, via secure email or another method of secure file sharing. Each image is a single digital photograph providing only one view of an area on the slide, which can limit diagnostic capabilities, especially if an important diagnostic focus is not captured. In dynamic telepathology, a “host” microscopist using a camera-equipped microscope streams video of the slide to the receiver, controlling all aspects of slide movement, objective selection, and focus. As an example, our institution has validated this method for sharing challenging intraoperative frozen section slides with colleagues in real-time. This technique depends on having a high-quality microscope camera. In addition, there must be good communication between the end-viewer and the person operating the microscope. Evaluating each slide using this method can be time consuming. Robotic telepathology shifts control of the stage, objective turret, and focus to the remote pathologist, who controls the microscope over a network connection. This method is less time consuming because the end-user has control over the microscope. However, this requires specialized equipment, including a microscope with motorized components and specialized software and controllers to manipulate the microscope remotely. In addition, there still needs to be a technician at the remote location who can load the slide into the robotic microscope. Whole-slide imaging creates a fully digitized, interactive image, which can be uploaded to a server and shared. The entire microscopic image is seen on the screen and can be viewed at different degrees of magnification. This method requires the availability of a whole-slide scanner, adequate file storage, and viewer software [11]. Importantly, all the above methods require skilled personnel to first perform the steps required to process tissue and generate an H&E-stained slide.

**Table 1:** Comparison of different telepathology methods

Modality	Workflow steps	Benefits	Drawbacks
Microscope camera and web-based image sharing (static and dynamic methods)	A technician manually drives the glass microscope slide and shows areas of interest to a remote pathologist. The pathologist interprets the image on a computer screen in real-time.	Cost-effective	Requires the microscope operator to have some histopathology knowledge for a smooth interaction and to show the relevant areas of the slide.
Robotic microscope with web-based capability for remote driving (robotic microscopy)	A technician loads the slide into the robotic microscope. A pathologist drives the slides remotely and provides interpretation.	Does not require the technician to have histopathology knowledge. Having control over the slide and microscope operation is more natural for pathologists.	Capital investment; hardware troubleshooting and repair; software management
Digitized image (whole-slide imaging)	A slide scanner creates a digital image of the glass slide, which is then uploaded to local or cloud-based storage and viewed remotely	Complete interpreter control Image can be stored to a system with a more reliable network connection	Capital investment; hardware repair; time needed to scan; requires IT infrastructure for data storage

The first commercial system for fully digital diagnosis, Phillips IntelliSite, was approved by the US Food and Drug Administration in 2017 [12]. A handful of scanners have now been FDA approved for diagnostic use, including Philips Ultra Fast, Hamamatsu NZ S360, and Leica Aperio AT2. These scanners each have a slide capacity of several hundred slides, with a scanning speed of 30–60 s per slide to digitize at the equivalent of 40x magnification [13], which is the highest-power objective most commonly used in routine surgical pathology. While the adoption of digital pathology for clinical use is steadily increasing in pathology departments across the United States, currently only a small minority of cases are diagnosed digitally [14].

Beyond digital diagnosis of H&E-stained slides of formalin-fixed paraffin-embedded tissues, several studies have investigated the feasibility of telepathology for intraoperative frozen section diagnosis [11, 15]. In general, the rate of post-washout intra-observer variability between digital and glass frozen section slides is low [11, 15, 16], suggesting that digital slide review does not sacrifice diagnostic accuracy. Interestingly, within GU pathology specifically, ureteral margin assessment for carcinoma was identified as a potentially disproportionate source of discordance, with the misdiagnosed digital slides tending to be false negatives (for example, missed urothelial carcinoma in situ in a ureteral margin) [17]. More studies are needed to identify specimen types that are especially prone to interpretation errors on digitized slides in order to find ways to mitigate them. Additionally, in current practice, generating and interpreting a digital image is significantly slower than traditional glass-slide methods in the intraoperative setting, with an average turnaround time estimated at 17 min per case for digital interpretation [17, 18]. While this falls within the recommended turnaround time range specified by the College of American Pathologists, the slower speed may be amplified in cases with multiple slides or multiple parts and should be addressed before full-scale rollout.

Implementation of digital pathology and telepathology, particularly for education, was accelerated out of necessity by the COVID-19 pandemic and has occurred in other times of natural disaster [17, 19]. To limit in-person interaction, teaching the evaluation and diagnosis of microscopic images went remote at many training institutions. In one workflow example, the pathology trainee would preview their assigned H&E glass slides. The corresponding faculty pathologist responsible for the case would then review the H&E slides with the trainee using remote meeting software such as Microsoft Teams or Zoom. In this case, the faculty pathologist would have control of the microscope, which is interfaced with a microscope camera, and the live video image and the glass slides would be shared with the trainee while discussing questions and teaching points.

Pandemic-induced government relaxation of Clinical Laboratory Improvement Amendments (CLIA) regulations enabled pathologists to finalize patient reports from a non-CLIA certified facility [20]. Under these updated regulations, a team from Memorial Sloan Kettering Cancer Center reported their experience and validation of their remote digital pathology system after digitizing 2119 glass slides (including 718 slides from GU cases), finding near 100% concordance between those cases signed out digitally and the glass slides subsequently re-reviewed in person [20]. Their study supports the noninferiority of WSI-based diagnosis even in the non-CLIA certified setting.

There are several barriers to implementing whole-slide scanning for routine diagnosis. Beyond the cost of the scanners themselves, which can cost in the hundreds of thousands of US dollars, institutions must also invest in substantial digital image storage capacity. At upwards of a gigabyte, digitized slide files can be orders of magnitude larger than digitized radiology images and require significant disk storage. Lastly, an efficient and reliable image management system is necessary for cataloging metadata and viewing the digitized slides. Despite these barriers, the benefits of digital pathology are numerous. They include reduced physical slide storage requirements, faster retrieval of archived materials, flexibility in slide review location, and leveraging software features such as built-in image analysis and measurement tools or the ability to view an H&E slide alongside registered special stains or IHC, without needing to switch slides on the microscope stage.

### The use of Digital Pathology for Expert Consultation

Consultation cases by their nature are considered challenging or ambiguous to interpret. Given this selection bias, these cases could be expected to stress-test the feasibility of digital review. Nonetheless, several studies have concluded that digital pathology for expert consultation is noninferior to glass slide-based consultation. Liu et al. provided expert head and neck pathologists with glass slides that they had previously diagnosed using digital pathology, after an extended washout period, and found minor discrepancies in only 5 of 57 cases, none of which reclassified a lesion as benign or malignant or would have changed clinical management [21]. No major discrepancies were identified. They concluded that given the low rate of minor discrepancies, digital pathology could supplant physical glass slides even in a population selected for difficult or challenging cases. In their study, the median turnaround time was approximately 70 h for a glass slide-based workflow, compared to less than 3 h for their digital workflow, with the transportation of glass slides identified as a significant rate-limiting factor. Additional studies have shown that digital consultation is efficient and accurate across multiple subspecialties [22–24].

### Telepathology in Global and Resource Limited Settings

Many rural areas have limited resources to perform quality pathologic examination of tissues removed from the body [19, 25, 26]. In order to provide a H&E-stained slide for primary diagnosis, tissue must undergo appropriate sampling, complex processing in hazardous chemicals, and often technically challenging cutting of formalin-fixed paraffin embedded tissues. The labor and technology associated with these processes is costly. In addition, once a slide is available for viewing, not all hospitals will have pathologists available to interpret the slide. It was estimated that in 2017 only 26% of low-income countries had pathology services available to the general public [25]. Even hospitals that have pathology services may not have subspecialty expertise for challenging cases and require expert consultation [26]. The ability to share digital histologic images can therefore be a powerful tool for pathologists around the globe. With the proper technology, specimens can be processed at a central location and images interpreted at separate locations with equivalent diagnostic accuracy compared to glass slides [23, 27].

Institutions from other countries have published on their real-world experiences utilizing digital pathology. A paper by He et al. evaluated the effectiveness of a cloud-based telepathology system in China [28]. Over a four-year period, 23,167 cases were evaluated, with 86% of cases originating from county-level hospitals. The use of telepathology was found to be diagnostically accurate and created cost savings of $300,000/year [28]. In Cameroon, the incidence of new cancer cases was 20,745 per year in 2020, with only 0.28 pathologists per 1 million people [29]. In 2018, the district hospital in Ngaoubela, which had no on-site pathologist, established a low-cost telepathology partnership with volunteer pathologists in Austria to provide tissue diagnoses. H&E glass slides were made in the district hospital’s histology lab from patient specimens and digitized, initially by an iPhone camera, and later by low-capacity slide scanner, and finally shared with the volunteer pathologists. Implementing the slide scanner reduced the rate of ambiguous diagnoses. The authors cited power supply interruptions as a major cause of delays in failures at several steps, impacting tissue processing and data transfer [29]. Saint Jean de Dieu Hospital in Tanguieta, Benin, receives patients from several surrounding countries such as Togo, Burkina Faso, and Nigeria. In 2015, this hospital opened a histology laboratory with telepathology capabilities [25]. The building and equipment were provided by the Galliera Hospital in Italy. Local laboratory technicians were trained for 9 months by pathologists from Europe in histology processing and digital scanning. Whole slide digital images were stored on a Web-accessible server and viewed by pathologists in other countries. From 2016 to 2018, 1593 patients had surgical specimens analyzed and 399 patients were diagnosed with cancer. The average turn-around-time from receipt of a specimen to a diagnosis was approximately 12 days [25]. Similar successes with digital pathology initiatives for low-income and middle-income countries such as Uganda [30], Afghanistan [31], Colombia [32], and other areas have been reported [33–38].

Although the potential global benefits of digital pathology can seem limitless, there remain obstacles for real-time implementation of this technology. While digital pathology allows for rapid sharing of stained slides, the consultant pathologist may want to perform additional workup in their own institution’s laboratory to reach a diagnosis, such as additional IHC stains or molecular testing. One option in this situation, if the tests are available in the referring pathologist’s lab, would be for the consultant pathologist to request that the submitting laboratory perform the desired workup and scan those slides for digital review together with the other parts of the case [21]. Otherwise, additional workup would require mailing physical material to the consultant pathologist, partially negating the benefits of a purely digital consultation. Furthermore, there can be a significant learning curve in pathologists transitioning from glass slides to whole slide imaging diagnostics [14, 23]. The implementation of digital pathology in other areas, such as in Latin America, faces many regulatory challenges, which is complicated by the current lack of standardization of the technology as well as the ability to provide digital data security. Although Brazil has specific regulatory requirements for telepathology, most other countries in Latin America lack national strategies for the implementation of this technology [39]. Additional challenges include training pathologists to adopt digital technology and the need for high-resolution scanners and viewing systems to provide high-quality images. There will also be increased costs involved in purchasing the technology as well as in the additional support staff required to maintain digital slide scanners [39]. Conversely, several low-cost alternatives have shown promise. Das et al.. reported their experience using an adapter to stream a live video feed of a microscope slide through the messaging platform WhatsApp [40]. Youssef and colleagues described their design of an affordable (less than 120 euros at the time of publication), portable microscope camera system to share a high-resolution live video stream [41]. Lastly, any lab that adopts digital pathology will require a workflow plan for cases of machine or server failures when telepathology is not available [42].

Artificial intelligence technology may also be of benefit in resource limited areas without adequate network infrastructure or subspecialist pathologist access. Prostate histology interpretation is one example where AI has recently been implemented with success. It is estimated that there will be nearly 300,000 new cases of prostate cancer in the United States in 2024 [43]. Several AI algorithms have been developed for the diagnosis and grading of prostate cancer [44]. Ibex’s Galen™ Prostate solution was the first AI-based cancer detection platform FDA-approved for clinical use [44]. A study by Santa-Rosario et al. evaluated the ability of this AI program to enhance diagnostic accuracy in an independent laboratory in Puerto Rico [45]. The authors found a 96.7% (95% CI 95.6–97.8) specificity and a 96.6% (95% CI 93.3–98.8) sensitivity, with a negative predictive value of 99.2% (95% CI 98.4–99.6). In theory, the results show the ability of the AI to help prevent missed cancer diagnoses while avoiding an overabundance of false-positive cores flagged as suspicious. Additional studies utilizing thousands of prostate biopsies were evaluated and the AI grade compared to experts in genitourinary pathology [46]. Studies showed that AI improved Gleason grading among pathologists and also improved diagnostic performance. With the assistance of an AI algorithm, pathologists demonstrated reduced interobserver variability. AI algorithms are currently being studied for their ability to not only diagnose and grade prostate cancer but also to evaluate risk for biochemical recurrence [46]. Other areas of investigation include predicting response to hormonal therapy and determining which patients are the best candidates for active surveillance [46].

## Conclusion

As cancer care becomes more and more individualized, the need for deep subspecialty domain knowledge for providing accurate histopathologic diagnosis continues to grow. However, shortages in the pathology workforce, especially in resource-limited areas, are an impediment to progress. Despite significant barriers to implementation, digital pathology and telepathology have the potential to leverage a global network of subspecialty pathologists to improve diagnosis and patient outcomes. The application of artificial intelligence techniques can further extend the capabilities of pathologists worldwide.

## Key References

• Patel AU, Mohanty SK, Parwani AV. Applications of Digital and Computational Pathology and Artificial Intelligence in Genitourinary Pathology Diagnostics. Surg Pathol Clin. 2022;15:759–85.

Provides a review of the state of digital and computational pathology in genitourinary pathology, with a focus on the role of artificial intelligence.

• Rohr JM, Ginnebaugh K, Tuthill M, Pimentel J, Markin R. Real-Time Telepathology Is Substantially Equivalent to In-Person Intraoperative Frozen Section Diagnosis. Arch Pathol Lab Med. 2023;148:68–73.

Describes a multi-year institutional experience with a real-time frozen section telepathology system, including remote diagnosis without an on-site pathologist. This study, one of the most comprehensive in terms of examined time frame and number of cases, found low rates of diagnostic discrepancies compared to traditional glass slides.

• Liu BL, Haghighi M, Westra WH. Digital Pathology is a Fast and Effective Platform for Providing Head and Neck Pathology Consultations. Am J Surg Pathol. 2024;48:985–90.

This study found high rates of concordance between digitized and glass slides in a head and neck pathology consultation practice, showing the noninferiority of digital pathology even after enriching for diagnostically challenging cases.

## Data Availability

No datasets were generated or analysed during the current study.
